# Subgraphs of Interest Social Networks for Diffusion Dynamics Prediction

**DOI:** 10.3390/e23040492

**Published:** 2021-04-20

**Authors:** Valentina Y. Guleva, Polina O. Andreeva, Danila A. Vaganov

**Affiliations:** National Center for Cognitive Research, ITMO University, 49 Kronverksky Pr., 197101 St. Petersburg, Russia; andreeva@itmo.ru (P.O.A.); vaganov@itmo.ru (D.A.V.)

**Keywords:** subgraph extraction, motif, sampling, dynamics prediction, social network, interest network

## Abstract

Finding the building blocks of real-world networks contributes to the understanding of their formation process and related dynamical processes, which is related to prediction and control tasks. We explore different types of social networks, demonstrating high structural variability, and aim to extract and see their minimal building blocks, which are able to reproduce supergraph structural and dynamical properties, so as to be appropriate for diffusion prediction for the whole graph on the base of its small subgraph. For this purpose, we determine topological and functional formal criteria and explore sampling techniques. Using the method that provides the best correspondence to both criteria, we explore the building blocks of interest networks. The best sampling method allows one to extract subgraphs of optimal 30 nodes, which reproduce path lengths, clustering, and degree particularities of an initial graph. The extracted subgraphs are different for the considered interest networks, and provide interesting material for the global dynamics exploration on the mesoscale base.

## 1. Introduction

Dynamical processes in different kinds of systems can be explored at a population level by means of continuous differential models, nevertheless, system organisation can be complicated by the non-regularity of interconnections between elements. This worsens the accuracy of population models built under the assumption of space homogeneity, and requires representation of interactions between elements by graph models.

The predictability of dynamics on graphs and building corresponding mathematical models is of great importance to overcome critical phenomena, resist crisis, and control systemic resilience to significant shocks. Despite large interest from research society, dynamics on graphs still leave topical due difficulties related to the discrete nature and changing heterogeneity of underlying space for process spread, which complicates measuring the conductance in a different part of a graph.

The variety of approaches concern this problem from different sides and scales. At a macrolevel, the correlation of spreading dynamics with graph topological properties is explored from a theoretical and empirical viewpoint. Namely, global topological features related to conductivity problems, epidemic thresholds for some kinds of generative models, equivalence of topological features from the dynamical point of view, simulations on different topologies, showing corresponding correlations, exploration of real networks, and dependence of their sensitivity to shock contagion on the underlying network structure. At a microlevel, the dynamics are related to node significance, local topological features, their effect on the spreading process, and significance measures development. The mesoscale is related to graphlets, motifs, samples, and other subgraphs, and is complicated by necessity of the subgraphs connection to the host graph from topological and functional properties. In this way, they can be interpreted as building blocks and can be used as one of the main descriptors [1]. Graph generative models can be partially related to a motif extraction problem since they search for a generative set, contributing to the graph formation process, which results in patterns observed in the final topology. Nevertheless, a certain graph is usually a noisy essence of some class, which is hard to process by contemporary available graph arithmetics.

Theoretical approaches to dynamics evaluation on the base of subgraphs are dependent on global network organisation and interactions between building blocks. In this way, approaches to Kronecker graphs, hierarchical networks, and block networks can be different. Approaches on the base of matrix factorisations or spectral graph theory involve theoretical issues of spectral relations between graphs and their part, which impede their direct application. In this way, understanding exploring network structures is important for the search and development of appropriate mathematical methods.

The structure of real networks, met in social, economical, and other human-made systems, contains much randomness due to uncertainty in decision making, but provide stable patters of link formation due to existing natural restrictions. In this way, stable building blocks associated with different kinds of systems seem to exists, and the addition of random noise and variation [2] results in observed entities. Therefore, initial building patterns are difficult to derive from behavioural patterns and observations due to systemic complexity. Another issue in subgraph extraction is its size due to (1) increasing computational costs with size due to the isomorphism problem and graph traversal, and (2) functional edge—a bigger subgraph contains more structures, making it similar to its host graph. This edge between functional quality, in terms of process spread, and motif size is explored in the current article in the context of social communication networks of different interests.

In this paper, we focus on the mesoscale exploration of dynamics on graphs. Our aim is to explore subgraphs of real networks, which distinguish them from each other and characterise functional properties from a dynamical viewpoint, and contrast how they look to the initial graph. In this way, we search for structures greater than motifs of 4–5 nodes to satisfy this functional property, but not too large for further interpretation and processing. In contrast, sampling methods used and compared for such purposes have been explored for much larger networks (5000+) extraction for crawling tasks, while here we need the smallest appropriate one.

For analysis we consider communication networks from Reddit related to different interests, and explore them in order to find (1) significant subgraphs specific to certain interests and (2) significant subgraphs having similar “conductance” to host graph relatively to the number of nodes. We also compare a comprehensive series of sampling techniques. Finally, we define a goodness criteria from the point of dynamics on graphs, and explore extracted subgraphs to be useful for dynamics prediction. As a result, we show the best subgraphs for different interest networks, and the most appropriate methods for their extraction. In this way, we contribute to the empirical exploration of social network building blocks, on the one hand, and explore the possibilities of dynamics estimation on the base of small subgraphs, on the other hand. Therefore, our results can be used further for the development of dynamics prediction methods on the basis of small subgraphs, and for the development of theoretical approaches for dynamics evaluation.

## 2. Related Studies

### 2.1. Correlation of Dynamics and Motif Structure—Empirical Studies

Natural sciences consider the effects of motifs on dynamics from the points of genes, molecules, cells, proteins, etc. at different scales, for example, the effects of molecule combinations and structures on energy levels and relaxation times [3]. They define functional or dynamic property and search for correlations between motif occurence and resulting property.

The contribution of motives to dynamics on networks is studied by [4], showing how motif abundance affects the structural stability score. They show the significance of a combination of motifs, having particular structural properties, with their frequency. In this way, the explored networks are divided into groups according to aggregated properties, which is observed for both, 3- and 4-node motives. Tan et al. [5] explore diffusion at individual and population scales in relation to motif structure and try to infer the diffusion network with the motif profile. Finally, diffusion networks are considered. Sarkar et al. [6] also use motifs to understand whether or not they can be used for an explanation of emerging cascades. For this, purpose edges, covered by the percolation algorithm, are compared with edges generated by motifs. In this way, existing studies, related to diffusion dynamics, are mostly focused on dynamic paths generated, and distinguish structural- and process-related motifs [7]. The majority of methods cover small motifs due to the high algorithmic complexity of this procedure and dynamics on whole networks being restricted by diffusion paths.

### 2.2. Analytical Methods for Dynamics Evaluation—Theoretical Studies

The estimation of synchronisation dynamics in large networks on the base of motifs [8] is provided by means of eigenvectors [9] of connection matrices, obtained from the initial motif by the Kroneker product. Lodato et al. [10] explore synchronisation dynamics for 3- and 4-node motifs, and analyse which of them are correlated with stability states (which looks quite similar to approaches from the previous subsection). D’Huys et al. [11] study Kuramoto oscillation models for three kinds of network motifs with different symmetries, and find numerical solutions for those single motifs. Nevertheless, this result is not expanded for the whole network. Motives synchronisation is also discussed in [12].

Synchronization patterns of Landau oscillators for hierarchical networks are explored from the point of spectral graph theory [8]. Authors obtain supergraph eigenvectors from their subgraph eigenvectors using spectral graph theory, and then incorporate them into solutions of dynamics equations at stationary points. In this way, the dependence of a global solution on local (namely, mesoscale) topological properties can be implemented via adjacency matrix replacement.

Chierichetti et al. [13] consider graph conductance and processes on graphs from an algorithmic viewpoint. Conductance is defined as graph cut in relation to the volume of the set being cut [14], and contagion is algorithmically approximated as a probability of node activation for a graph of considered conductance.

The derivation of global network dynamics from local connectivity patterns and higher order motives are explored in [15] using a theoretical framework, cumulating inputs and outputs of motives. In this way, the study can be connected to renormalisation group approach [16], but with a focus on inner and integral dynamics instead of geometric decomposition.

### 2.3. Subgraph Extraction Techniques: Motives, Samples, and Communities

Subgraph extraction is often aimed at understanding global network properties through their components and interaction structure. In this way, laws invariance for different scales is supposed, which can be a formal criteria for graph partitioning success evaluation [1]. Benson et al. [17] aim at finding a frequent structural block and try to minimise summarised conductance—graph cut via motives. In this way, resulting structures do not intersect and represent clustered organisation.

From the structural point of view, renormalisation group theory supposes the presence of isomorphic structural patterns at different scales [18]. They suppose a generative set with repeating symmetries as a building block for the modelling of network formation by means of group theory [18]. In practice, they implement node embeddings of different dimensions into a supernode and decompose real networks in a self-similar way.

Motives, samples, and communities are notions met in graph partitioning tasks, differentiated by the following particularities: Motives are met often, so they are proposed to be building blocks, communities demonstrate higher density inside and can be of different sizes, and samples aim at global graph properties reproduction.

Motif is a subgraph or a pattern in a larger graph which is statistically significant. Statistical significance means that the pattern appears in a given graph more often than it would in a random graph of the same size and degree distibution. There are some measures [19] of pattern frequency based on restricting overlapping subgraphs. The F1 measure counts all possible occurrences of a pattern even if elements are used several times. F2 and F3 measures are more restictive, because they count only those subgraphs which do not share any edges (F2) or even any nodes (F3). Methods that search patterns based on F2, F3 measures are said to mine edge-disjoint or node-disjoint motives in a graph.

Most of the methods of mining disjoint motives are covered in [20]. All of them are based on the same concept of constructing overlap graph and finding the Maximum Independent Set (MIS) on it. Each node in the overlap graph denotes a motif in the given graph. There is an edge in the overlap graph between two nodes if corresponding motives are overlapping in the target graph.

As soon as the MIS problem is NP-hard, methods usually use heuristics to find the approximate size of the MIS.

For example there are two methods: HSIGRAM and VSIGRAM [21], which employ a breadth-first or depth-first search strategy to generate from subgraphs of size k cndidates for larger subgraphs of size (k + 1). After generating all motives, F2 or F3 measures of every pattern is countered by two ways. First, the exact is searching the maximum clique of a complementary graph to a target and for the second, the approximate strategy uses one of the most widely-used heuristic—the greedy algorithm. This heuristic selects a node from the overlap graph of the minimum degree and deletes this node with all of its neighbours and the procedure is repeated.

Authors of this paper show that there is no significant difference between exact and greedy algorithm in run-time nor in measures. The only minus of the methods is that they assume that the network is undirected, sparse, and labelled.

There is a tool called MAVisto [22] that implements a Frequent Pattern miner algorithm (FPF) [23]. FPF uses a method that builds a tree similar to the idea of HSIGRAM, but it applys downard closure property, which states that the frequency (F2, F3) of patterns is monotonically decreasing with the increasing size of the pattern [19]. Due to this property not all of the branches of the tree are used. Besides, FPF allows parallelisation, so it is faster than previous methods. For counting the MIS, the greedy algorihm is used.

Another approach to mining disjoint motives is GREW [24], which works in an iterative fashion, merging certain subgraphs that are connected to each other via one or multiple edges. GREW is shown to be faster than VSIGRAM, but it is not guaranteed to find all the vertex-disjoint motives.

One paper [25] shows that all the connected motifs with more than three edges can be constructed by joining four basic patterns of size 3. So the MIS of these four patterns is identified using the greedy algoritm. Then, the algorithm counts only the edge-disjoint motives.

SuperNoder [26] uses the FANMOD [27] tool to find all motives in the graph. Then it proposes five heuristics to find the MIS, compared to all other methods, which uses only the greedy algorithm for this purpose. FANMOD is based on the RAND-ESU [28] algorithm that enumerates all nodes and the building tree. Enumerating nodes allows one to break the overcounting of one motif. This method is faster than MAVisto, but MAVisto supports counting the F3 measure inside the tool and SuperNoder is needed to count the MIS separately from FANMOD.

### 2.4. Building Blocks of Real-World Networks

Huang et al. [29] explore biological networks for brick and bridge motives, different in strength of ties, which is related to density notion, while their occurence in networks corresponds to global clusterisation. Brain networks were analysed for 3- and 4-node directed motives [30] and demonstrated different frequency spectra with a prevailing path and V-structure for the 3-node case.

Colomer-de-Simón et al. [31] comprehensively explore real networks structural components, distinguishing k-cores, triangles, modules, and hierarchical organisation, resulting (propositionally) from local opimisation rules.

Fundamental structures of real-worlds was shown to be correlated with their “control profiles” [32]. Structure is explored in terms of flow theory and dominating control patterns, considering sources and sinks. In this way, different kinds of real networks are displayed by different heat-maps (no exact motifs or any sub-graphs).

García-Pérez et al. [18] apply geometrical renormalisation to different real networks using components of size 2, and then compare them in the space of the power law degree distribution exponent and renormalisation exponent. This results in two-dimensional hyperbolic representation of networks geometry, which corresponds to building blocks of generative sets being equivalent to embedded subgraphs. Geometrical scaling is also related to multi-fractal networks characterisation and their structural complexity and heterogeneity [33].

All these approaches seems to restrict their search for small motif sizes, which are usually not enough for social networks demonstrating high variability. Here we continue the exploration of interest networks [34] and search for larger subgraphs, reflecting the structural and dynamical properties of a supergraph.

## 3. Materials and Methods

The article explores the building blocks of social networks under the invariance of their dynamical properties. In this way, we aim to find the smallest subgraphs, demonstrating diffusion dynamics similar to a corresponding supergraph. The final goal is to see the extracted subgraphs for different types of social networks and explore if they are appropriate for the prediction of dynamics for the whole network, i.e., they have the appropriate size and demonstrate similar topological and functional (dynamical) properties.

**Figure 1:**
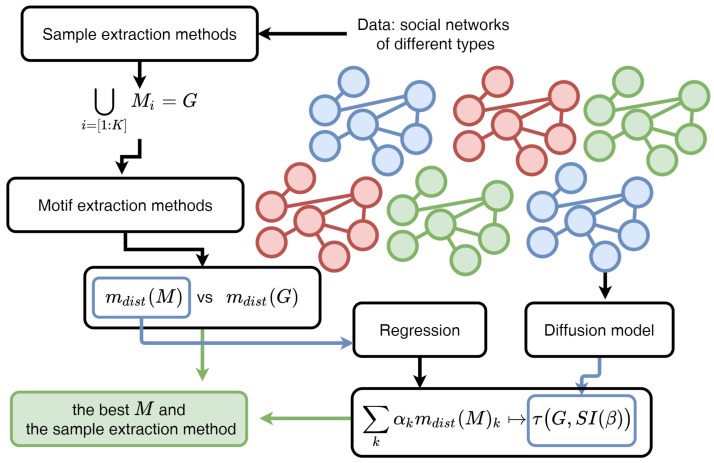
The framework of the appropriate subgraphs search. Sampling methods are used for subgraph extraction, then motif extraction techniques are used for topological verification: Motif distributions for sub- and supergraphs are compared; the regression model on the base of sample motif distribution is used for transient time prediction for the SI diffusion model. The best subgraphs and corresponding methods for topological and functional criteria are evaluated.

This section formalises the problem statement and desired properties of extracted subgraphs, and then briefly describes the subgraph extraction methods and data considered, which are combined together into the graph processing framework (Figure 1), comprising topological and functional correspondence components.

The graph structure is supposed to be relatively homogeneous to have a representative subgraph inside, which seems to be possible under the proposition it was formed by laws and connection patterns, related to a considered group type. To connect graph structure with dynamics, we describe a diffusion process on a graph by a population model with discrete node states (susceptible-infected dynamics), and connect diffusion times for initial graph and its subgraphs, which is the consequence of their “consuctance” equivalence. In other words, the existence of a representative subgraph, which determines spreading dynamics on the system as a whole, is supposed.

In more details (Figure 1), the data graphs $G_{i}$, corresponding to single topics, are processed by different sampling techniques, which maps a set of subgraphs $\left\{M_{i}\right\}$ to each graph. Then, subgraphs are compared with initial graphs pairwise by their motif distributions, performed by motif extraction methods. This stage implements topological comparison and indicates the best sampling techniques in a topological viewpoint. Dynamical view point indicates the best sampling methods for dynamics prediction, evaluating the number of iterations of diffusion on graphs on the base of a subgraph motif distribution.

### 3.1. Problem Statement

Consider a system of state *S*, structure of element interaction *G*, and dynamic process D(S,G,t), which describes changes of system state *S* with time *t*. The goal is to characterise by a constant η(G,D) systemic ability to conduct the process D using a smaller subgraph M⊂G, which is supposed to demonstrate the same dynamical properties η(M,D) against the considered process D, i.e., η(M,D)≈η(G,D).

To clarify the proposition, let dynamics on a graph be defined by the Susceptible-Infected (SI) epidemic model [35]. Then, in a general case, it can be said nodes are activated or not, which means there are two types of node states at a micro-level: $s_{i}\in \left\{0;1\right\}$, which affects the system state as $S=\frac{\sum s_{i}}{\#V(G)}$. For the full graph and infection rate *r* of the SI process, the change in *S* is described by the well-known equation, modified for the case of varied number of neighbours in a graph with arbitrary topology:

(1) $$\frac{dI}{dt}=C\left(G\right)rI\left(N-I\right).$$

Then, for the Erdos–Renyi graph effects of graph structure on process spreading (let us say graph conductivity C(G)) are related to density, while graphs with higher modularity have higher neighbour probability in a module than outside. Due to beliefs in network formation patterns, we suppose the existence of a subgraph with similar average density, which contributes to dynamics on a graph with the same intensity:

(2) ∃M⊂G:C(M)≈C(G),

i.e., C(M) allows for supergraph characterisation, due to dependence in its formation patterns (or structure of interactions) and node states.

Thus, system state at a macro-level depends on conductivity at a mesoscale, determining the number of active neighbouring nodes, and the solution of Equation (1) allows to evaluate the number of iterations τ(G) for a system to reach an active state from an initial one. Then for block network structure Equation (3) is supposed to be true:

(3) $$\tau \left(M\right)∼\frac{\#M}{\#G}\tau \left(G\right),$$

where τ is trasient time for a graph to come from an initial state to an active one.

i.e., the desired subgraphs are invariant and relatively activating times considering their sizes, while the objective methods allow for extraction subgraphs of such invariance.

### 3.2. Resulting Subgraph Verification

Several methods are going to be explored in the frame of current research, therefore, the algorithm for their tuning and result verification is required.

#### 3.2.1. Topological Similarity

According to Equation (2), topological similarity of global properties is necessary for extracted subgraphs. In order to estimate it from a formation patterns angle and since motives seems to reflect main the topological features, we compare motif distributions of sub- and supergraphs, and evaluate mean squared deviation for these sequences.

Consider graph fragmentation into *K* samples, then for each sample there exists a motif distribution:

(4) $$\begin{array}{c} G=\underset{i\in [1:K]}{⋃}M_{i},\;\;\underset{i}{⋂}M_{i}=\emptyset , \end{array}$$

(5) $$\begin{array}{c} \forall M_{i}\in \underset{i\in [1:K]}{⋃}M_{i}{\mapsto R|}_{\left[0;1\right]} \end{array}$$

(6) $$\begin{array}{c} m_{dist}:G\to \left\{P\left(M_{i}\right)\right\}. \end{array}$$

Here, $m_{dist}$ is a motif distribution, and $P(M_{i})$ is a probability of $M_{i}$-th motif in a *G* graph. Then,

(7) $$\delta \left(M,G\right)=\sum_{k}{\left(m_{dist}{\left(G\right)}_{k}-m_{dist}{\left(M\right)}_{k}\right)}^{2}$$

is a measured correspondence between the *M* motif and supergraph *G* motif distributions.

#### 3.2.2. Regression Model for Transient Time Prediction

In order to provide equivalence between dynamic properties of a graph and its subgraph, proposed in Equation (3), we build a regression model predicting trasient time τ(G) on the base of its subgraph features. It is proposed proportional to $\tau (M_{i}^{j})$,where *i* is a sample identifier and *j* is a sampling technique.

Each extracted subgraph is characterised by the set of motives, and the predictive regression model is built on their base. In this way, predicted transient time for the *G* graph is mapped to extracted subgraph properties. Therefore, for each method *j* and corresponding subgraph $M_{j}$ with motif distributions $m_{dist}\left(M_{j}\right)$, where *j* iterates over motif extraction methods, the regression model is:

(8) $$\forall \sum_{jk}\alpha_{jk}f\left(m_{dist}{\left(M_{j}\right)}_{k}\right)\to \tau \left(G\right),$$

where τ(G) empirically evaluated over 10 runs of the SI epidemic process, and $\alpha_{jk}\in R$ are tuned.

The learning process was organised as follows: In order to obtain explainable results by means of the SHAP library, CatBoost regression [36] was chosen. The model was used with default parameters, except number_of_iterations=100. The 5-fold cross-validation was performed using the ShuffleSplit method from the SKlearn python library, where test size was always set to 30%. As a prediction quality measure, the coefficient of determination ($R^{2}$) was used, s. t. the resulting $R^{2}$ was averaged among all folds.

In this way, the predictive ability on the base of extracted subgraphs is evaluated. Motif and sample extraction techniques are described further in the article.

### 3.3. Subgraph Extraction Techniques

Methods of subgraph extraction are aimed at discovering possible ways of real networks organisation with attention to their functional properties. This section describes the particularities of methods chosen for exploration in this article and comprises methods, extracting dynamically representative subgraphs (Sampling Methods, Section 3.3.1) and auxiliary methods for their topological comparison (motif extraction methods, Section 3.3.2).

#### 3.3.1. Sampling Methods

The sample extraction stage is aimed at finding graph building blocks, reflecting functional (in our case, dynamical) properties of a supergraph.

A large number of methods are implemented in littleballoffur package [37], but in this work we chose various methods that aimed to show the best results in the experiment of estimating descriptive statistics on social-network data sets. Among them, there were methods on the basis of local node properties, edge properties, and process-based methods using random walk implementation.

Node-based techniques select a set of representative vertices according to a chosen distribution, for instance uniformly (RN) [38] or according to the pre-calculated PageRank score of the vertices (PRN) [39]. The second step is extracting the induced subgraph.

Edge-based methods randomly select subset of edges by sampling those uniformly (RE) [40].

Another big group of sampling techniques is exploration-based. It includes breadth-first search, depth-first search strategies, and random walk. Snow Ball sampling (SB) [41] is a modification of BFS that uses a fixed number of neighbours. Community Structure Expansion sampling (CSE) [42] is a local greedy method as it samples nodes from which it can reach the greatest number of unknown nodes. Shortest Path sampling (SP) [43] add nodes to a sample and a random shortest path between them, including nodes and edges on this path.

Remaining methods induce a subgraph from nodes that participated in a random walk [44]. Metropolis–Hastings Random Walk (MHRW) [45] eliminates the problem of bias towards visiting high degree nodes in a graph by making the walker visit lower degree nodes.

The drawback of stucking the walker in a closely-knit community is solved by several methods such as the Random Walker Jumps (RWJ) [46], which teleports from a current node to a random one with fixed probability or non-backtracking random walk, where steps backwards are restricted [47].

#### 3.3.2. Motif Extraction Methods

This class of methods is auxiliary and aimed at a comparison of the smallest building blocks of networks as main topological representatives. They are applied to extracted samples and a supergraph for further comparison of correspondence.

The SuperNoder [26] algorithm with light modifications was chosen for counting motives. As a first step, it counts the number of all motives of a specific size by enumerating all nodes and building the tree. Each child node of this tree is an extension of its parent. It includes the vertices from the initial graph, that the parent contains, and a neighbour of this set whose number is greater than all others vertices. One modification is the separation of motives into groups of isomorphism. Thus, the different types of motives are distinguished. On the second step, the heuristic of greedy eliminations is done to find disjoint motives. For this purpose, all motives are shuffled and each motif is added to a MIS, if it is not overlapping all other instances in this set. Otherwise it is removed.

Tree variations of motif frequencies are considered:

- The ratio of every type of motif to a total number of all motives of the same size;
- The ratio of every type of disjoint motives to a total number of all motives of the same size;
- The ratio of all disjoint motives of the exact size to all motives of this size.

### 3.4. Data Set

The Reddit data, focused at discussions around a number of topics, were used for analysis. (Update of the Reddit corpus from 2018: https://www.reddit.com/comments/8aen5g, accessed on 20 April 2021 [48].) Each topic was modelled by 11 graphs, corresponding to each month of discussions observed. Each graph contains nodes corresponding to people, and edges representing their answers during discussions, related to posts. In this way, people communicating with each other are connected by edges.

The initial data set was processed by building corresponding networks and evaluating their topological properties. Graph sizes, density, global clustering coefficients, and degree-degree correlations were analysed. The limits of observed topological properties were analysed and corresponding topics were chosen to provide a comprehensive variety of explored properties (Table 1).

Topics from the table were further explored in the article.

## 4. Results

Initially, topologically similar subgraphs were extracted by several sampling techniques (Section 4.1), which then were checked for their functional correspondence (Section 4.2) by means of regression models. Subgraphs and the most appropriate methods are presented.

### 4.1. Topologically Similar Subgraphs

Here we show how the best subgraphs look for social networks of different interests and demonstrate which methods allow for their extraction. The topological difference from the supergraphs are demostrated by means of MSE.

All methods extract samples which get closer to an initial subgraph with an increase in size, which is natural for statistical reasons. At the same time, the results clearly distinguish methods on the base of processes and random walks as showing the smallest MSE and fast convergence to their minima. The most similar subgraphs to the initial networks were obtained (sample size was >20) by Community structure expansion, random walk, non-back tracking random walk samplers, and Metropolis-Hasting. This is similar to the result on the base of disjoint motifs, showing Metropolis-Hasting, random walk, non-back tracking random walk, and community structure expansion samplers. The weakest results in terms of topological similarity are presented in the degree and edge samplers, and Page-Rank-based method.

**Figure 2:**
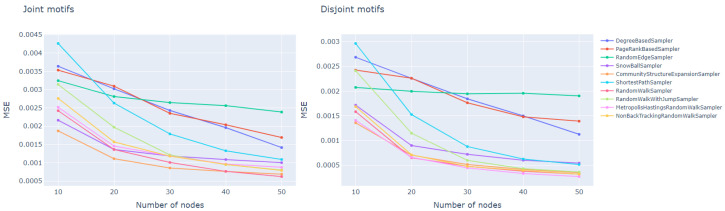
Mean squared error between motif distribution of extracted subgraphs and corresponding supergraphs for different sampling techniques.

For disjoint motifs, MSE shows lower values for all sizes of samples, varying from 0.0014–0.003 (for 10 nodes) to 0.00025–0.0019 (for 50 nodes), which is on average two times lesser than for the joint motif case, varying from 0.002–0.0043 (for 10 nodes) to 0.0007–0.25 (for 50 nodes) (Figure 2).

Since the aim is not to find the largest subgraphs, but just more representatives in a topological and dynamic viewpoint than motifs of four nodes, the subgraphs of 30 nodes look satisfactory and interpretable for visual analysis.

### 4.2. Methods for Significant Subgraphs for Dynamics Prediction

From the process viewpoint, regression models on the basis of joint and disjoint methods provide similar results. At the the same time, other sampling methods lead in quality.

**Figure 3:**
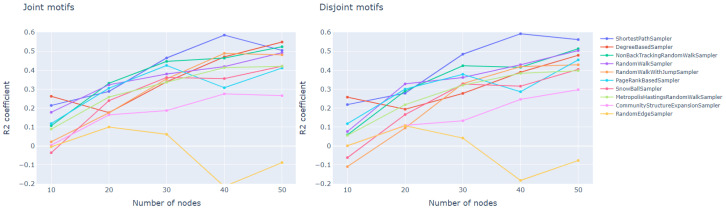
Coefficient of determination for regression model over subgraph *M* motif distribution, predicting τ(G,D) for different sampling techniques.

The best prediction is reached for the 40-node subgraph by the shortest path sampler, and for the 50-node subgraph by the degree-based and non-back tracking random walker. For the disjoint motif case, the shortest path sampler still demonstrate the highest quality for 30–50 node samples, and for the 50-node samples non-back tracking and random walk samplers stay at 2nd and 3rd position. Other samplers provide prediction accuracy (R2) below the value of 0.5 (Figure 3).

### 4.3. The Best Functional Subgraphs of Social Networks

**Figure 4:**
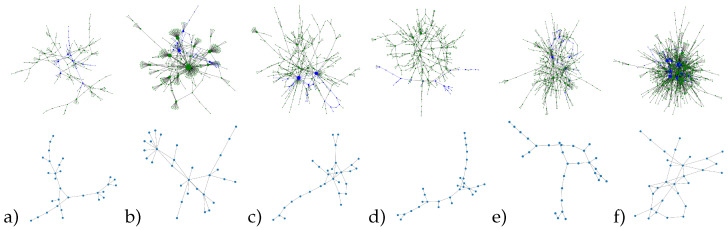
Resulting subgraphs on the corresponding supergraphas and their single versions for the selected topics: (**a**) Counter strike, (**b**) Free donut—i like it!, (**c**) feminism, (**d**) introvert, (**e**) pizza, and (**f**) stop smoking. Node size correspond to its degree, blue nodes highlight subgraphs extracted.

From the previous sections the top sampling techniques were obtained from the point of topological closeness to initial networks and their ability to be used for dynamics prediction. Generally, methods showing less difference in motif distributions between graph and its subgraph, demonstrated worse prediction accuracy, and vice versa. Nevertheless, non-back tracking random walk sampler was the first method met in both tops. In this way, we selected it for further exploration. A variation of subgraph sizes shown, that it is 30 nodes, after which changes in the MSE and R2 become significantly lesser, therefore, the extracted subgraphs were of 30 nodes (Figure 4).

The results show that the non-back tracking random walk sampler extract subgraphs, repeat topological patterns, and allow to distinguish different networks from each other. One can see the path length patterns, cycle patterns, hubs, and relative density. For the donut network, having more visible hubs than other networks (node size correspond to its degree), we see a pair of hubs in the subgraph, in contrast to prevailing long paths in introvert and pizza groups. The stop-smoking group, having the densest connections, has higher clustering and cycles in the extracted subgraph.

## 5. Discussion and Future Work

The extracted subgraphs contain the main building blocks of an initial graph, which affect the spreading process from a global viewpoint. They contain path, clustering, and degree features, which results from the first condition about topological similarity, and comparison of subgraphs by motif sequences. In this way, the chosen method fits this requirement better by experimental construction. By theoretical knowledge, the same topological features are responsible for process spreading, which explains this method fitness to the second functional condition.

In this way, the results seem to reproduce the dynamics of process spreading and can be used for predictive and analytical models of dynamics. Practical use of a method is restricted by its computational complexity, which is actually not high, and more effective than motives evaluation. In addition, methods on the basis of random walks do not always require a consideration of the whole graph, which is faster than the simulation of a process. Therefore, for future research it would be interesting to build initial graph traversal with complexity proportional to subgraph size.

The lack of results is that the extracted subgraphs still look unordered, and not connected to any formation law with clear generative sets, which can be a consequence of high variability of social data. In addition, the justification of the observed subgraphs may be questionable. Despite argumentation, presented in the Methods section, the expected form of resulting graphs was not clear initially, and one of the goals of this study was to see them and their relation to initial graphs. Now, after obtaining these results, we seem to be closer to building connection between dynamics on different subgraphs, and to develop prediction models for large graphs on the base of their subgraphs. The future work in this direction is to understand how subgraphs differ and how to process them further to connect with dynamics on supergraphs. This will be useful for the theoretical application of extracted subgraphs to dynamics prediction.

The results look very promising and demonstrate small structural components of social networks of different interests, and corresponding method, allowing for their fast extraction with no lack of structural and dynamic properties. From the algorithmic viewpoint, future research on the base of current results can include building a graph traversal method with exit criteria for determining host graph conductance on the base of subgraph. In this case, subgraph size is unknown in advance, and we prefer to avoid the whole graph processing. In this way, current results about sample size reflecting dynamical properties of the whole graph seems to be necessary.

In general, the network structure is complicated by node states. Therefore, the connection between dynamics at a meso- and macro scale will allow a generalised multiscale dynamics model, involving node states at a microlevel to be built.

## 6. Conclusions

The article explored building blocks of social networks, which are able to reflect topological and functional properties. For this purpose we developed a formal method of data processing on the basis of justification from processes on graphs formalism. Then we compared different sampling techniques and obtained appropriate sizes of subgraphs (30 nodes), showing enhancement in topological and functional correspondence. Finally, we demonstrated the extracted subgraphs and their place on the supergraphs.

## Figures and Tables

**Table 1:** The discussion topics finally chosen and the corresponding graph topological features (features are averaged for several networks within topics of interests).

	Nodes	Clustering	Density	Assort.
badtattoos	703.(36)	0.0071	0.0018	−0.0506
gonewildcurvy	1191.(54)	0.0064	0.0015	−0.1482
southpark	2031.(81)	0.0109	0.0008	−0.0236
HogwartsRP	54.(09)	0.3462	0.1432	−0.2259
redditblack	201.(72)	0.3055	0.0453	−0.1748
geology	554.(54)	0.0214	0.0025	−0.0541
hardwareswap	1712.(27)	0.0613	0.0025	−0.0818
counterstrike	446.(81)	0.0086	0.0025	−0.0668
stopsmoking	830.(63)	0.0503	0.0024	−0.1102
memes	443.(27)	0.0142	0.0023	−0.0313
feminism	675.(36)	0.0259	0.0022	−0.0310
introvert	581.(90)	0.0145	0.0022	−0.0863
pizza	615.(54)	0.0198	0.0021	−0.0882
vegetarian	804.(27)	0.0266	0.0021	−0.0594
depression	2973.(09)	0.0139	0.0005	−0.0726
CrazyIdeas	2600.(63)	0.0127	0.0005	−0.0488
lifehacks	3126	0.0119	0.0004	−0.0635
conservatives	126.(18)	0.2698	0.0191	−0.4898
90daysgoal	169.(27)	0.4219	0.0545	−0.4184
csshelp	253.(54)	0.0526	0.0067	−0.4451
freedonuts	605.(27)	0.0228	0.0026	−0.3823
altgonewild	112.(72)	0.0075	0.0111	−0.3164
bonsai	318.(45)	0.3263	0.0128	−0.3188
colorado	464.(90)	0.0289	0.0036	0.04103
GreenBayPackers	1576.(18)	0.0317	0.0019	0.0159
beertrade	806.(72)	0.0413	0.0034	0.0100

## Data Availability

Publicly available data sets were analysed in this study. This data can be found here: update of the Reddit corpus from 2018, https://www.reddit.com/comments/8aen5g (accessed on 20 April 2021).
